# Platelets Selectively Regulate the Release of BDNF, But Not That of Its Precursor Protein, proBDNF

**DOI:** 10.3389/fimmu.2020.575607

**Published:** 2020-11-25

**Authors:** Jessica Le Blanc, Samuel Fleury, Imane Boukhatem, Jean-Christophe Bélanger, Mélanie Welman, Marie Lordkipanidzé

**Affiliations:** ^1^ Faculty of Pharmacy, Université de Montréal, Montréal, QC, Canada; ^2^ Research Center, Montreal Heart Institute, Montréal, QC, Canada

**Keywords:** platelets, brain-derived neurotrophic factor, secretion, plasma, pro-BDNF

## Abstract

**Background:**

Brain-derived neurotrophic factor (BDNF) plays a role in synaptic plasticity and neuroprotection. BDNF has well-established pro-survival effects, whereas its precursor protein, proBDNF, induces apoptosis. Thus, it has been suggested that the proBDNF/BDNF ratio could be an indicator of neuronal health. Access to neurons is, understandably, limited. Because of their similarities, platelets have been put forward as a non-invasive biomarker of neuronal health; indeed, they store large quantities of BDNF and can release it into circulation upon activation, similarly to neurons. However, whether platelets also express the precursor proBDNF protein remains unknown. We therefore sought to characterize proBDNF levels in human platelets and plasma.

**Methods:**

The presence of proBDNF was assessed by immunoblotting, cell fractionation, flow cytometry, and confocal microscopy in washed platelets from 10 healthy volunteers. Platelets from 20 independent healthy volunteers were activated with several classical agonists and the release of BDNF and proBDNF into plasma was quantified by ELISA.

**Results:**

Platelets expressed detectable levels of proBDNF (21 ± 13 fmol/250 x 10^6^ platelets). ProBDNF expression was mainly localized in the intracellular compartment. The proBDNF to BDNF molar ratio was ~1:5 in platelets and 10:1 in plasma. In stark contrast to the release of BDNF during platelet activation, intraplatelet and plasma concentrations of proBDNF remained stable following stimulation with classical platelet agonists, consistent with non-granular expression.

**Conclusions:**

Platelets express both the mature and the precursor form of BDNF. Whether the intraplatelet proBDNF to BDNF ratio could be used as a non-invasive biomarker of cognitive health warrants further investigation.

## Introduction

The brain-derived neurotrophic factor (BDNF) is a member of the neurotrophin family initially identified in the central nervous system where it is known to play a role in synaptic plasticity, long-term memory, cognition and neuroprotection (1–3). It is produced as a precursor protein, proBDNF, which is then cleaved by intracellular or extracellular proteases to release the pro-domain and the mature BDNF protein (2, 4). Like BDNF, proBDNF can be released by neurons following an action potential (5, 6), and has an active biological function, which is in opposition with the pro-survival functions of BDNF. Indeed, proBDNF induces neuronal apoptosis (5), reduces dendritic arborization (6), and negatively regulates synaptic plasticity (6, 7) and transmission (6).

The contrast between the role of BDNF and that of its precursor has led to the hypothesis that the regulation of the proBDNF/BDNF ratio is important for maintenance of a healthy nervous system (4, 5, 7, 8). In line with this hypothesis, it has been shown that the regulation of proBDNF cleavage is a key process in long-term hippocampal synaptic plasticity (9) and in memory (10, 11). Furthermore, some authors suggest that impairment in proBDNF cleavage could be important in the pathophysiology of cognitive disorders (12, 13). Several studies report a change of the proBDNF/BDNF ratio in neurons or cerebrospinal fluid in diverse neurocognitive disorders such as Alzheimer’s disease (13, 14), major depressive disorder (8), autism (15), and affective disorders (4).

Although it was at first discovered in the brain, we now know that BDNF is also present in blood, where it is essentially stored in platelets (16, 17). BDNF levels in platelets can reach 100- to 1,000-fold those of neurons, making platelets the most important peripheral reservoir of BDNF (17–20). Similarly to neurons, platelets store BDNF mainly in α-granules (21) and release it into the bloodstream during platelet activation (16). While proBDNF has also been reported in circulation (22, 23), the origin of peripheral proBDNF is unknown. It is particularly puzzling, as the presence of proBDNF in platelets has never been confirmed.

We therefore sought to investigate the presence of proBDNF in platelets; to compare the plasma *vs*. platelet levels of proBDNF in healthy volunteers; and to investigate the release profiles of proBDNF *vs*. BDNF in response to platelet activation induced by different agonists. As several studies have suggested that platelets could be a potential biomarker of neuropsychiatric disorders, this study could open up new avenues of research on the intraplatelet proBDNF/BDNF ratio as a biomarker of neurocognitive health (24–28).

## Materials and Methods

### Participant Selection

This study was approved by the Montreal Heart Institute Scientific and Research Ethics Committee (REC reference: #2016-1996) and all participants gave written informed consent. A total of 30 participants were included in this study; in 10 subjects, platelets were isolated and washed to better characterize the presence of proBDNF in platelets, and in 20 subjects, proBDNF and BDNF levels were quantified in platelets and plasma following platelet activation. Participants were exempt of chronic diseases, did not require chronic medical treatment, had refrained from drugs influencing platelet function in the 2 weeks preceding blood sample collection, and had normal platelet count and hemoglobin levels. Participants with a history of bleeding were excluded.

### Blood Collection and Platelet Isolation

Using a 21G needle, blood was drawn by venipuncture into 30 ml syringes containing either acid citrate dextrose (ACD-A) in a 1:5 volume ratio for experiments carried out in washed platelets, or sodium citrate 3.2% in 1:9 volume ratio for experiments in platelet-rich plasma (PRP). Blood samples were gently mixed by inversion, transferred to 50 ml tubes and centrifuged at 200 g for 15 min without a brake to prepare PRP and at 1,000 g for 10 min to prepare platelet-poor plasma (PPP).

Native citrated PRP was used without adjustment of platelet count for platelet aggregation experiments, with autologous PPP used to set baselines.

To obtain washed platelets, prostaglandin E_1_ (1 µM) was added to ACD-anticoagulated PRP prior to centrifugation at 1,000 g for 10 min. Platelets were resuspended in Tyrode’s buffer (137 mM NaCl, 11.9 mM NaHCO_3_, 0.4 mM NaH_2_PO_4_, 2.7 mM KCl, 1.1 mM MgCl_2_, 5.6 mM glucose, pH 7.4). This washing procedure was repeated three times. Platelets were counted using a Beckman Coulter hematology analyzer (Ac-T 5diff AL) and adjusted to a final concentration of 2.5 x 10^8^/ml for flow cytometry experiments or 2 x 10^9^/ml for cell fractionation and deglycosylation experiments. Platelets were allowed to rest at room temperature (RT) for 60 min prior to functional experiments.

### Cell Fractionation

Washed platelets (2 x 10^9^/ml) resuspended in phosphate buffered saline (PBS) were lysed by three freeze-thaw cycles (temperatures of −80 and 37°C). The samples were then centrifuged at 200,000 g for 90 min at 4°C. The supernatant representing the cytosolic fraction was transferred to a new tube, while the pellet representing the cytoskeleton and membranes was solubilized in radioimmunoprecipitation assay (RIPA) buffer [150 mM NaCl, 5 mM EDTA pH 8.0, 50 mM Tris-HCl pH 8.0, 1% NP40, 0.5% sodium deoxycholate, 0.1% sodium dodecyl sulfate (SDS)] and incubated on ice for 15 min to dissolve the membranes. The solubilized sample was then centrifuged at 100,000 g for 30 min at 4°C. The supernatant in which the membranes were dissolved was transferred to a new tube, while the pellet representing the cytoskeleton was resuspended in 1X Laemmli buffer and heated at 95°C for 5 min with vigorous vortexing. Samples were then assessed by immunoblotting using antibodies against proBDNF (as described below), BDNF (Biosensis, M-1744, monoclonal mouse antibody, 1 μg/ml), p-selectin (Santa Cruz, sc-6941 polyclonal goat antibody, 0.26 μg/ml), α-tubulin (Sigma-Aldrich, T5168 monoclonal mouse antibody, clone B-5-1-2, 1.225 μg/ml), and p65 NF-ĸB (Santa Cruz, sc-372 polyclonal rabbit antibody, 0.4 μg/ml). The equivalent in the protein content of 3 x 10^7^ platelets was loaded for each fraction on the gel.

### Deglycosylation

Washed platelets and U87-MG cells were lysed with ice-cold RIPA buffer for 20 min. Platelet or U87-MG cell lysates (100 μg) were denatured at 100°C for 10 min in a glycoprotein denaturating buffer (0.5% SDS, 40 mM DTT, B1704S, New England Biolabs, MA, USA) and allowed to cool to RT. GlycoBuffer 2 with 1% NP-40 (New England Biolabs, MA, USA) was added to denatured proteins, and PNGase F (P0704S, New England Biolabs, MA, USA) was then added to a final concentration of 50 units per µg of total protein. The mixture was incubated at 37°C for 1 h, frozen and kept at −80°C. Immunoblotting was performed using antibodies against proBDNF (Biosensis, R-176, polyclonal rabbit antibody, 0.25 μg/ml), CD42b (Santa Cruz, sc-59051, monoclonal mouse antibody, clone PM6/40, 1 μg/ml), or sortilin/NT3 (Abcam, ab16640, polyclonal rabbit antibody, 2 μg/ml). The equivalent of the lysate of 3 x 10^7^ platelets was loaded in each well.

### ProBDNF and α_2_-Macroglobulin Immunoblotting

Human recombinant proBDNF (Alomone Labs, Israel) and Human Brain Cerebral Cortex Whole Tissue Lysate (Novus Biologicals, Bio-Techne, Oakville, ON, Canada) were used as positive controls. Native platelet whole cell lysates were incubated in Laemmli loading buffer (250 mM Tris pH 6.8, 8% SDS, 40% glycerol, 20% β-mercaptoethanol, and 0.02% bromophenol blue) before heating at 95°C for 5 min. Protein samples were separated on a 12% SDS-polyacrylamide gel electrophoresis (PAGE), transferred onto a PVDF 0.2 µm membrane and fixed with glutaraldehyde 0.5% for 30 min. Membranes were washed three times with TBS-T (Tris-buffered saline, 0.1% Tween 20) for 10 min and blocked for 1 h at RT in blocking solution [3% bovine serum albumin (BSA) in TBS-T or 5% milk in TBS-T] before incubation with anti proBDNF or anti α_2_-macroglobulin antibodies (Biosensis, anti-proBDNF R-176 polyclonal rabbit antibody, 0.25 μg/ml; R&D systems, anti-proBDNF mab31751 monoclonal mouse antibody, clone 584412, 0.5 μg/ml; Abbexa, anti-α_2_-macroglobulin abx132389, monoclonal mouse antibody, 1 μg/ml) overnight at 4°C. The blots were then washed and incubated with HRP-conjugated goat anti-rabbit or goat anti-mouse secondary antibody (Jackson ImmunoResearch Laboratories, diluted 1:10,000 in 5% milk) for 1 h at RT. Luminata Classico Western HRP Substrate (Millipore) was used for chemiluminescent detection.

### Flow Cytometry

Washed platelets (2.5 x 10^8^/ml), U87-MG or U251-MG cells were fixed with 1% paraformaldehyde (PFA) for 20 min at RT, a fraction of which was also permeabilized using 0.1% Triton-X for 15 min at RT. Platelets and neuroblastic cells were then incubated at RT with mouse anti-human proBDNF antibody (R&D System, mab31751, monoclonal mouse antibody, clone 584412, diluted 1:25 or Biosensis, R-176 polyclonal rabbit antibody diluted 1:25) or mouse IgG_2b_/rabbit isotype control (R&D System, MAB004 and AB-105-C diluted 1:25) for 30 min. Alexa Fluor 488 or 647 conjugated donkey anti-mouse or rabbit secondary antibody (Invitrogen, diluted 1:100) was added for 30 min at RT in the dark. Samples were analyzed using the MACSQuant Analyzer 10 (Miltenyi Biotec, Germany).

### Confocal Microscopy

Glass coverslips were pre-coated with 0.1% poly-L-lysine for 15 min at RT. Platelets fixed with 1% PFA for 20 min at RT were transferred onto pre-coated coverslips and allowed to adhere overnight at 4°C. Fixed U251-MG cells were plated on the pre-coated coverslips and incubated overnight at 37°C/5% CO_2_ incubator. Platelets and U251-MG cells were permeabilized using 0.1% Triton X-100 in PBS for 10 min at RT. Coverslips were then washed twice with PBS and blocked with 3% donkey serum in PBS for 30 min at RT. Coverslips were then washed two times with PBS and proBDNF labelling was performed using an anti-proBDNF primary antibody (R&D, mab31751, monoclonal mouse antibody, clone 584412, 5 µg) for 2 h at RT followed by two PBS washes and an incubation with an Alexa Fluor 488-conjugated donkey anti-mouse secondary antibody (1:200) for 90 min at RT. Mouse IgG_2b_ was used as isotype control antibody (R&D System, MAB004, clone 20116, 5 µg). After two more washes, U251-MG coverslips were incubated with diluted DAPI (10 mg/ml, 1:1,000) for 5 min and washed again twice with PBS. Coverslips were then treated with 1,4-diazabicyclo(2,2,2)octane (DABCO) mounting medium (25 mg/ml DABCO in 90% glycerol/10% PBS solution) overnight in the dark. Fluorescence was visualized using a Zeiss LSM510 confocal microscope.

### Light Transmission Aggregometry

Platelet aggregation was measured using a Chronolog Aggregometer (Model 700, Havertown, PA, USA) at 37°C with continuous stirring at 1,200 rpm. Platelet aggregation traces were recorded for 6 min using the AGGRO/LINK^®^8 Software package. The following agonists were used: adenosine diphosphate (ADP, Sigma Aldrich) 10 μM, arachidonic acid (AA, Cayman Chemical) 1 mM, collagen (Chronolog) 5 μg/ml, or thrombin-related activating peptide (TRAP-amide, Bachem) 3 μM. A vehicle-treated PRP sample was used under stirring conditions as a control. Ethylenediaminetetraacetic acid (EDTA, 5 mM) was added at the end of the incubation to stop platelet activation and agitation was continued for 1 min. Platelets and plasma were separated by centrifugation at 1,000 g for 5 min and placed into separate tubes. Platelets were lysed for 30 min on ice with lysis buffer (1% NP40, 20 mM Tris pH 8.0, 137 mM NaCl, 10% glycerol, 2 mM EDTA, 1 mM sodium orthovanadate) containing Pierce Protease and Phosphatase Inhibitor Mini Tablets (Thermo Scientific). Lysed platelets and supernatants containing the platelet releasate were kept frozen at −80^o^C until analysis.

### Assessment of BDNF and proBDNF Levels

Plasma and intraplatelet concentrations of BDNF and proBDNF were determined by ELISA (R&D System, DY248 and DY3175). Plasma and platelet lysate samples were centrifuged at 3,000 rpm for 5 min to remove cellular debris. Samples were diluted 1:15 in reagent diluent (**Supplementary Figures 1** and **2**). Assays were performed in accordance with manufacturer’s instructions. Cross-reactivity for mature BDNF was negligible (1.4%) in the proBDNF assay, and low (13%) for proBDNF in the BDNF assay. Each condition was tested in duplicate. Colorimetric reading was performed with the Infinite F50 plate reader (Tecan, Männedorf, Switzerland) at 450 nm, with a reference at 620 nm.

### Statistical Analyses

Continuous variables are presented as mean and standard deviation (SD) or median and interquartile range (IQR) when distribution deviated from normal. N refers to the number of independent experiments with each experiment representing a different biological sample. Repeated-measures analysis of variance (ANOVA) with Geisser-Greenhouse correction for sphericity and Dunnett’s correction for multiple comparisons was performed to assess differences between agonist-stimulated conditions. Pearson correlation was used to explore the association between proBDNF and BDNF levels. All analyses were carried out with GraphPad Prism Software version 8 for MacOS (GraphPad Software, San Diego, CA, USA). A multiplicity-adjusted p value < 0.05 was considered significant.

## Results

### Human Platelets Contain proBDNF

**Figure 1:**
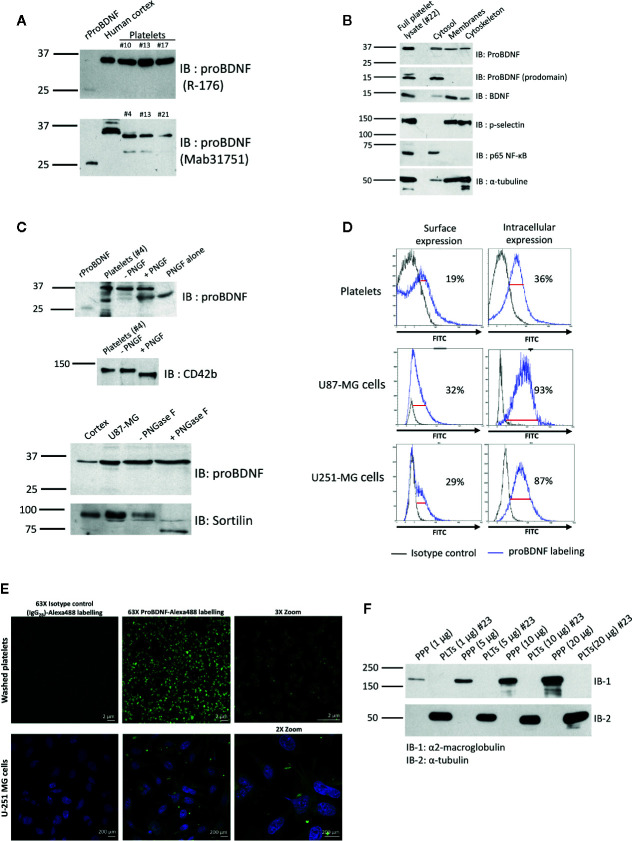
Human platelets contain proBDNF. **(A)** ProBDNF immunoblotting of human washed platelet lysates (15 µg) from six different healthy volunteers. Recombinant proBDNF (3 ng) and human cortex lysate (3 µg) were used as positive controls. Molecular weight is indicated on the left (kDa) and primary antibody on the right. Experiments representative of n=9 for R-176 and n=10 for mab31751 antibody. IB, immunoblotting. **(B)** Immunoblotting of proBDNF and BDNF in different fractions of washed human platelets. P-Selectin was used as control protein in the membrane fraction, p65 NF-ĸB was used as control protein in the cytosol, and α-tubulin was used as control protein in the cytoskeleton. The equivalent of the protein content of 3 x 10^7^ platelets was loaded for each fraction on the gel. Representative experiment of n=4 different volunteers. **(C) **ProBDNF treatment with PNGase F in washed human platelet lysates. U87-MG glioblastoma cells were used as a control. rProBDNF, recombinant proBDNF (3 ng); cortex, human cortex lysate (3 µg); platelets, whole human platelet lysate (representative experiment of n=4 different volunteers, 7.5 x 10^8^ platelets per well); −PNGF, platelets treated with GlycoBuffer, and incubated at 37°C for 60 min without PNGase F; +PNGF, platelets treated with GlycoBuffer and incubated at 37°C for 60 min with PNGase F; PNGF alone, PNGase F incubated at 37°C for 60 min without platelet lysate. CD42b and sortilin were used as controls of protein deglycosylation in platelets and in U87-MG cells, respectively. n=3 different volunteers for PNGase treatments in human platelets and n=4 independent experiments for U87-MG cells. **(D)** Representative flow cytometry experiment showing surface and intracellular proBDNF in human washed platelets and in U87-MG and U251-MG glioblastoma cell lines. Mouse IgG_2b_ was used as isotype control. Percentage of expression are indicated on the figure. n=10 different healthy volunteers for human platelets; n=3 independent experiments for each glioblastoma cell line. **(E)** Confocal microscopy imaging of proBDNF in human permeabilized washed platelets (top) and in permeabilized U-251 MG cells (bottom). Mouse IgG_2b_ was used as isotype control. ProBDNF was labelled using Alexa488 fluorochrome (in green). Nuclei were stained with DAPI (in blue). Scale bar = 2 µm and 200 µm for washed platelets and U-251 MG cells images, respectively. **(F) **Immunoblotting of α_2_-macroglobulin at increasing quantities of loaded proteins (1–20 µg) obtained from a washed platelet lysate or platelet-poor plasma (PPP) from the same individual (#23). α-tubulin was used as loading control. Molecular weight is indicated on the left (kDa) and primary antibody on the right. PPP, platelet poor plasma; PLTs, platelets; IB, immunoblotting.

To assess the presence of proBDNF in platelets, we performed immunoblotting experiments on washed platelets using two different proBDNF antibodies (**Supplementary Figure 3**). To verify that detection of proBDNF in platelets was not due to plasma protein contamination, we cross-checked for immunoreactivity for α_2_-macroglobulin in platelet lysates (**Figure 1F**), showing absence of this plasma protein in the washed platelet preparation. Human cortex used as positive control, expressed proBDNF at ~32–35 kDa (**Figure 1A**). Recombinant proBDNF produced in *Escherichia coli* was detected at a lower molecular weight (~25–27 kDa). In platelets, we detected a band at ~32–35 kDa (**Figure 1A**). Following incubation with PNGase F, a clear shift was seen for CD42b, a platelet membrane glycoprotein used as a positive control for N-deglycosylation (**Figure 1C**). Although a lower band did appear below the 32–35 kDa band for proBDNF, the same band was also visible in the absence of platelet protein, likely due to non-specific binding to PNGase F (expected molecular weight 36 kDa). Treatment of U87-MG human glioblastoma cell lysates with PNGase F also failed to show N-deglycosylation of proBDNF. This suggests that the differences in molecular weight between platelet and neuronal BDNF *vs*. recombinant proBDNF were not attributable to N-glycosylation.

### Platelet proBDNF Is Localized in the Cytoplasm

The cytosolic, membrane (including granular membranes), and cytoskeletal fractions of platelets were obtained by differential ultracentrifugation and analyzed by immunoblotting. To confirm the cellular compartmentation of the fractions, we used NF-κB as a marker for the cytosolic fraction, P-selectin for the membrane fraction, and α-tubulin for the cytoskeletal fraction (**Figure 1B**). Mature BDNF was mainly found in the membrane fraction (presumably in α-granules), but also at lower levels in the cytosolic and cytoskeletal fractions (**Figure 1B**). In contrast, proBDNF was distributed similarly in each of the three fractions (**Figure 1B**). Interestingly, a lower band around the 15-kDa marker which could correspond to the cleaved pro-domain of proBDNF (29, 30), exclusively segregated into the cytoplasmic fraction (**Figure 1B**).

Immunoblotting for α-tubulin showed immunoreactivity in the membrane and cytosol fractions, suggesting suboptimal cytoskeletal fraction separation. Therefore, to further investigate proBDNF localization, we performed flow cytometry and confocal microscopy on fixed and permeabilized platelets. A mean of 13 ± 8% of platelets expressed proBDNF at their surface, and 40 ± 20% were positive for proBDNF once cells were permeabilized (**Figure 1D**). To validate that detection of proBDNF was not due to plasma protein adsorption on platelet plasma membranes, acid-washed platelets were compared with platelets washed in Tyrode’s buffer at physiological pH, with no significant drop in proBDNF signal in acid-washed platelets (**Supplementary Figure 4**). Confocal microscopy imaging also suggested that proBDNF was present at the membrane and in the cytosol of platelets (**Figure 1E**). Human glioblastoma U87-MG and U251-MG cells used as positive controls showed mainly intracellular expression by flow cytometry, and microscopy further confirmed that expression was essentially cytosolic and nuclear (**Figures 1D, E**).

### Human Platelets Contain Less proBDNF Than BDNF

**Figure 2:**
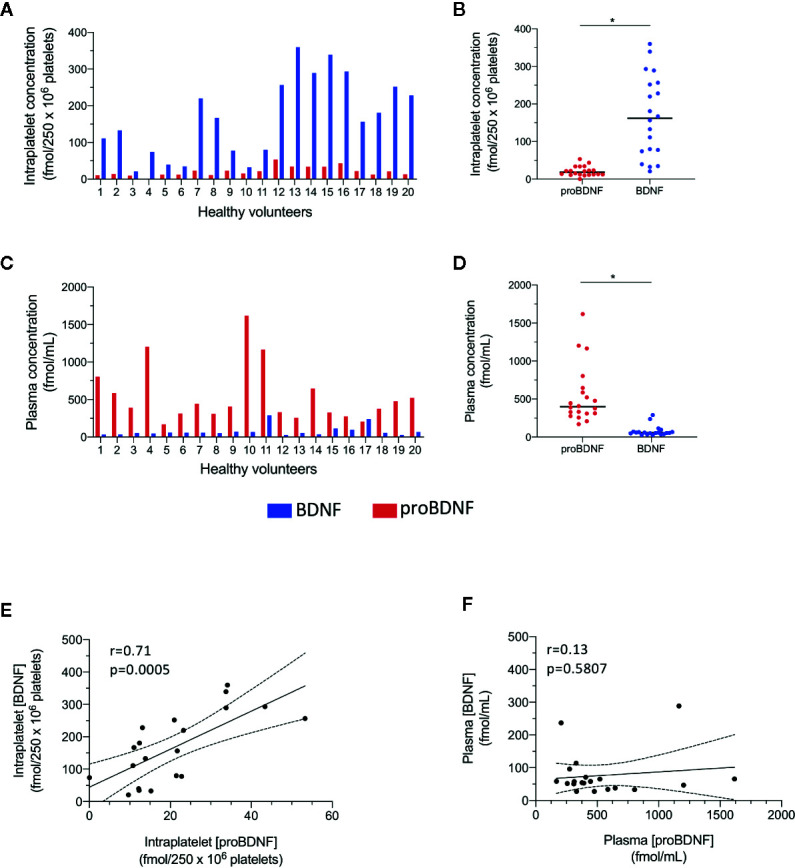
Molar concentrations of proBDNF are lower in platelets and higher in plasma than those of BDNF. ELISA quantification of proBDNF and BDNF levels in the intraplatelet **(A, B)** and plasma **(C, D)** compartments. Concentrations are normalized for 250 x 10^6^ platelets. Horizontal bar represents median, *p<0.05. **(E, F)** Correlation between BDNF and proBDNF molar concentrations in human platelets **(E)** and in plasma **(F)**. Dotted lines represent 95% confidence intervals, n=20 participants (#1 to #20).

To assess the relative abundance of proBDNF and BDNF in platelets, we quantified both proteins by ELISA in 20 healthy volunteers (**Table 1**). The levels of proBDNF were variable from one individual to the other (**Figure 2A**), but under basal conditions, proBDNF levels were significantly lower than BDNF levels (proBDNF 1,085 ± 672 pg/250 x 10^6^ platelets equivalent to 21 ± 13 fmol/250 x 10^6^ platelets compared to 4,516 ± 2,915 pg/250 x 10^6^ platelets equivalent to 167 ± 108 fmol/250 x 10^6^ platelets for BDNF). The mean intraplatelet proBDNF/BDNF molar ratio was 0.18 ± 0.14 (**Figure 2B**), meaning that there was ~1 molecule of proBDNF for ~5 molecules of BDNF in platelets (**Table 2**).

**Table 1:** Characteristics of the healthy volunteers included in the ELISA quantification study (n = 20).

	Number of participants, n (%)
**Sex**	
Female Male	13 (65) 7 (35)
**Age (years)**	
20–29 30–39 40–49 50–59	9 (45) 4 (20) 5 (25) 2 (10)
**Ethnicity**	
French Canadian North African Caribbean	18 (90) 1 (5) 1 (5)
**Body mass index (kg/m^2^)**	
<18.5 (underweight) 18.5–24.9 (normal weight) 25.0–29.9 (overweight) 30.0–34.9 (class I obesity) 35.0–39.9 (class II obesity) >40.0 (class III obesity)	1 (5) 10 (50) 8 (40) 1 (5) 0 (0) 0 (0)
**Smoking status**	
Smoker Ex-smoker Non-smoker	0 (0) 3 (15) 17 (85)
**Daily physical activity level**	
Sedentary Light Moderate Vigorous	4 (20) 1 (5) 15 (75) 0 (0)

**Table 2:** proBDNF/BDNF ratio as a function of platelet activation status.

	Platelet state	Mean proBDNF/BDNF ratio (n=20)	SD	95% CI	p-value
**Intraplatelet**	**Basal**	0.18	0.14	0.12–0.24	
	**Activated**				
	ADP (10 μM)	0.46	0.33	0.31–0.60	0.0003
	TRAP (3 μM)	0.56	0.43	0.37–0.75	0.0003
	AA (1 mM)	0.60	0.37	0.43–0.76	0.00001
	Collagen (5 μg/ml)	0.59	0.43	0.40–0.78	0.0002
**Plasma**	**Basal**	10.2	7.9	6.7–13.7	
	**Activated** ADP (10 μM)	2.5	4.3	0.60–4.4	0.0001
	TRAP (3 μM)	2.3	3.6	0.70–3.8	0.00005
	AA (1 mM)	3.6	7.4	0.38–6.8	0.003
	Collagen (5 μg/ml)	2.4	3.1	1.02–3.8	0.0002

### Plasma Concentrations of proBDNF Are Higher Than Those of BDNF

We then investigated whether a similar proBDNF/BDNF ratio was found in circulation. Under basal conditions, we found the opposite pattern in plasma to that observed in platelets (**Figure 2C**), i.e., concentrations of proBDNF in plasma were much higher than those of BDNF (proBDNF 28,019 ± 19,695 pg/ml or 541 ± 380 fmol/ml compared with BDNF 2,064 ± 1,825 pg/ml or 76 ± 68 fmol/ml). We calculated the mean proBDNF/BDNF ratio to be ~10 molecules of proBDNF for ~1 molecule of BDNF in plasma (**Figure 2D**, **Table 2**).

### BDNF and proBDNF Concentrations Are Correlated in Platelets But Not in Plasma

**Figures 2E, F** show the association between intraplatelet and plasma levels of proBDNF and BDNF. While a linear correlation was seen between BDNF and proBDNF in platelets (r=0.71; p=0.0005, **Figure 2E**), no such association was seen in plasma (r=0.14; p=0.58, **Figure 2F**), suggesting the regulation of proBDNF/BDNF ratio is different in the cellular and plasma compartments.

### Unlike BDNF, proBDNF Is Not Released During Platelet Activation

**Figure 3:**
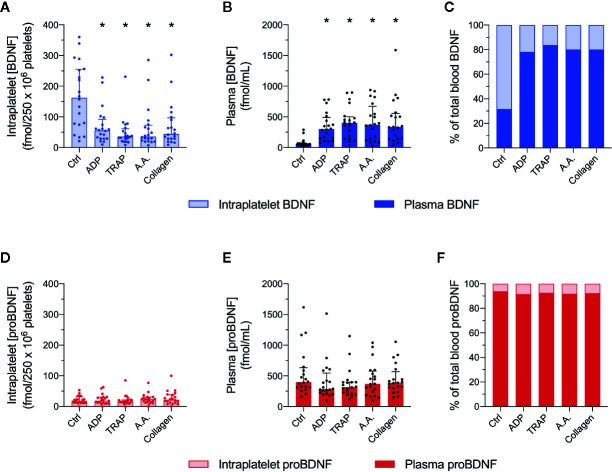
Unlike BDNF, intraplatelet proBDNF is not released during platelet activation. Intraplatelet **(A, D)** and plasma **(B, E)** concentrations of BDNF and proBDNF following platelet activation by different agonists. Intraplatelet concentrations are normalized for 250 x 10^6^ platelets. Proportion of BDNF **(C)** and proBDNF **(F)** in plasma *vs*. in platelets are expressed in percentage. Error bar represents IQR, *p < 0.05 *vs*. ctrl, n=20 participants (#1 to #20). Ctrl, control; ADP, adenosine diphosphate; TRAP, thrombin receptor-activating peptide; AA, arachidonic acid.

We next studied whether platelets have the ability to release proBDNF in the same manner they release BDNF during their activation. Platelet responses to four different platelet agonists (ADP, TRAP, AA, and collagen) were assessed in platelet-rich plasma from 20 healthy volunteers. As expected, intraplatelet concentrations of BDNF decreased with the addition of platelet agonists and plasma concentrations increased, confirming that BDNF was released from platelets during their activation (**Figures 3A, B**). As shown in **Figure 3C**, resting platelets contained ~70% of total BDNF present in PRP, and this proportion decreased to ~20% after platelet activation.

In contrast, intracellular and plasma levels of proBDNF remained stable in response to platelet activation (**Figures 3D, E**). Only ~10% of proBDNF in PRP appeared to be stored in platelets, regardless of platelet activation status (**Figure 3F**). As a consequence, platelet activation significantly changed the proBDNF to BDNF ratio in plasma from 10:1 to close to 2:1 (**Table 2**).

## Discussion

In the present study, we have shown that: 1) platelets contain proBDNF in a 1:5 ratio to BDNF; 2) the levels of intraplatelet proBDNF correlate strongly with those of BDNF, whereas no such association was seen in plasma; 3) the pool of intraplatelet proBDNF represents approx. 10% of total circulating proBDNF, whereas approx. 70% of circulating BDNF is stored in platelets; and 4) platelet activation does not lead to proBDNF secretion, in contrast to BDNF that is largely released following platelet activation.

### Presence of proBDNF in Platelets

Since the early 1990’s, numerous reports have conclusively shown platelets to contain large quantities of BDNF as to represent the major peripheral reservoir of this neurotrophin (16, 17, 31, 32). Circulating proBDNF, on the other hand, has received considerably less attention. While it is known that proBDNF is produced by many cell types including neurons (5, 6), megakaryocytes (18), lymphocytes (33), skeletal muscle (34), and endothelial cells (35), the contribution of these cells to circulating proBDNF levels is not elucidated.

To our knowledge, there is only one group that has investigated the presence of proBDNF in platelets (18). Chacón-Fernández *et al.* have shown platelet precursor cells, megakaryocytes, to express proBDNF, but failed to detect proBDNF in mouse, rat, and human platelets with an antibody targeting the mature BDNF protein (18). With the use of antibodies targeting the prodomain of proBDNF, we were able to show that platelets do contain proBDNF, albeit in a much lesser proportion to its mature counterpart. The origin of BDNF in platelets remains debated. Chacón-Fernández *et al.* have shown BDNF to be present in megakaryocytes, their proplatelet extensions, and in platelets, suggesting BDNF is inherited from megakaryocytes (18). Fujimura *et al.* have shown platelets to internalize exogenous BDNF, suggesting they may acquire it from the bloodstream (16). The relative contribution of inherited *vs*. internalized BDNF in platelets remains unknown.

Our results show a high variability in circulating BDNF and proBDNF levels among healthy individuals. While the parameters influencing proBDNF circulating levels have not been the object of investigation, several factors are known to affect blood levels of mature BDNF, including age (36–39), sex (37–39), smoking status (37), and body mass index or weight (38, 39). Our small sample size precludes multivariate analyses to explore the contribution of these characteristics to circulating levels of proBDNF/BDNF in this study, but this should be explored in larger cohorts. Attention should also be given to ethnicity, as it appears to be an important determinant of circulating proBDNF levels (40).

Notwithstanding, for the 20 individuals tested, we found significantly less proBDNF than BDNF in platelets, resulting in a mean 1:5 ratio of proBDNF to BDNF. This ratio is in line with levels observed in the central nervous system, where the proBDNF/BDNF ratio was 1:10 in hippocampal cells (29, 41). However, while proBDNF was shown to be N-glycosylated in human neurons (42) and saliva (43), the platelet proBDNF did not appear to be sensitive to PNGase F treatment, thus arguing against N-glycosylation in these cells. It should be noted however that PNGase treatment of U87-MG glioblastoma cells also failed to induce changes in molecular weight, suggesting that other post-translational modifications may explain the differences of molecular weight with recombinant proBDNF produced in bacteria (25–27 kDa). Five different isoforms of proBDNF have been repertoried in the literature, each with a different molecular weight (44–46). Whether differences in molecular weight seen in this study are due to different isoforms expressed or different post-translational modifications of proBDNF in megakaryocytes and platelets requires further investigation.

In stark contrast to intraplatelet levels, plasma levels of proBDNF were 10 times those of BDNF. This is surprising, in view of the short half-life of BDNF in circulation (47, 48) suggesting that proBDNF is protected from degradation in plasma. It has been shown that mature neurotrophins including BDNF bind reversibly to α_2_-macroglobulin (49), a plasma protease inhibitor and transporter, protecting them against proteolytic degradation and clearance pathways (50). Whether proBDNF can similarly bind to α_2_-macroglobulin and thus be protected from proteolytic cleavage, or uses a different mechanism to avoid degradation, is worthy of investigation.

### Not All Platelets Express proBDNF

Our flow cytometry experiments showed that a mean of 13 ± 8% of platelets had proBDNF on their surface and 40 ± 20% were proBDNF-positive upon permeabilization. Although platelets underwent gentle washing procedures to avoid platelet activation, we can not eliminate the possibility that some of the proBDNF found on the platelet surface is in fact residual from plasma. However, no plasma protein contamination was detected in our platelet lysates (**Figure 1F**), and acid washing to remove plasma proteins adsorbed on cell membranes did not reduce the proBDNF signal in platelets (**Supplementary Figure 2**), thus limiting this possibility. We observed a high level of correlation between intracellular BDNF and proBDNF levels, and found a proBDNF fragment of approx. 15-kDa consistent with the proBDNF pro-domain in the platelet cytosol, both suggesting that an intracellular regulation mechanism of the proBDNF/BDNF ratio is present in platelets, or is vestigial from their precursor megakaryocytic cells and inherited by platelets during thrombopoiesis. In neurons, proBDNF cleavage is processed by furin, metalloproteinases (MMP-9) (51, 52), and protein convertases (PC1, PC5, PACE4, and PC7) (4). Platelets express several proteases that could possibly participate in BDNF maturation, such as furin-like proprotein convertases (53) and other proteases stored in platelets granules such as MMP-2 and MMP-9 (54). Thus, it is conceivable that proBDNF cleavage occurs in platelets in a regulated manner. While the body of evidence on the role of the pro-domain in the nervous system is growing, it was undetected for many years (55), and its characterization required a complex optimization of techniques (29). In the peripheral nervous system, the cleaved pro-domain is 10-fold more abundant than proBDNF, and both the pro-domain and BDNF are secreted from the same synaptic intracellular vesicles (29). The pro-domain appears to contribute to regulation of neuronal growth, and to essential mechanisms of depression and psychological disorders (30, 56, 57). Therefore, a thorough investigation of the presence and the potential role of the pro-domain in platelets is warranted.

### Unlike BDNF, proBNDF Is Not Released Upon Platelet Activation

Our results confirm that platelets release approximately 50% of their BDNF content during activation. Tamura *et al*. have made the same observation and found that there are two distinct pools of BDNF in human platelets: a releasable pool of BDNF stored in α-granules and a non-releasable pool of BDNF localized in the platelet cytoplasm (21). However, Tamura *et al.* used an antibody raised against the mature portion of BDNF, and therefore could not distinguish between the precursor and mature proteins. In our experiments using antibodies raised against the proBDNF pro-domain, we have found a significant proportion of proBDNF to be present in the cytoplasm, as well as in the membrane and cytoskeletal fractions, which might explain the absence of release upon platelet activation. Considering that intraplatelet proBDNF represents only approximately 10% of total circulating proBDNF, and that platelets do not release significant levels of proBDNF upon activation, it is unlikely that plasma proBDNF comes from platelets.

Several groups have explored the possibility that plasma proBDNF is neuronal in origin. However, while it has been suggested by some authors that mature BDNF might cross the blood-brain barrier in mice and rats (58, 59), this finding was not supported by others (47, 48), and none have specifically investigated the permeability of the blood-brain barrier to proBDNF. Thus, the origin of the high levels of proBDNF seen in plasma remains to be elucidated. Notwithstanding, platelet activation induces a dramatic change in the plasma proBDNF to BDNF ratio, by releasing large quantities of mature BDNF (**Table 2**). Any future use of plasma proBDNF to BDNF ratio as a potential biomarker for neurocognitive health will thus need to take into account platelet activation status.

### Limitations

Although we were able to show proBDNF in human platelets by three different techniques, there are limitations that require pause. First, the platelet release experiments were performed in plasma, which naturally contains BDNF and proBDNF. Thus, it is possible that the experimental model does not allow the detection of a weak release of proBDNF. Second, we have added EDTA at the end of the platelet activation experiments to inhibit calcium-dependent proteases. We cannot exclude the possibility that proBDNF was rapidly cleaved into BDNF following platelet activation and thus might not be measurable in the supernatant. However, intraplatelet proBDNF levels remained unchanged during platelet activation, which lends credence to the fact that there is no detectable proBDNF release upon platelet activation and argues against the two previous limitations. Our cell fractionation experiments suggest that proBDNF is equally present in cytoplasmic, membrane and cytoskeletal fractions. We used a crude technique for separation of cell fractions, with good resolution of the cytoplasmic from the membrane fraction, but with residual contamination from the cytoskeletal fraction. These results should therefore be interpreted alongside flow cytometry and microscopy assessments. Finally, it is possible that proBDNF and BDNF bind to plasma proteins, which might mask epitopes from detection by ELISA. However, both stimulated and control samples were handled in the same way, and the rise of BDNF levels was readily detectable in plasma. Thus, it is unlikely that release of proBDNF was missed in agonist-stimulated *vs*. resting platelets.

## Conclusions

To our knowledge, this study is the first to report the presence of proBDNF in human platelets. Granted we do not provide the certainty of sequencing through mass spectrometry, but within the limitations of our assays, we are confident that what we are seeing is indeed proBDNF in platelets. It seems however unlikely that platelets contribute to a significant extent to circulating proBDNF levels, since only ~10% of the total circulating levels of proBDNF were found within platelets. Furthermore, in contrast to BDNF, platelets did not release proBDNF during activation, reinforcing the likelihood that the high levels of proBDNF observed in plasma originate from another cell type. The correlation between BDNF and proBDNF concentrations within platelets leads us to hypothesize that there is an intracellular mechanism regulating the proBDNF/BDNF ratio, albeit it could be vestigial from megakaryocytes. Further studies are required to elucidate the role of proBDNF in platelet function and the contribution of the intraplatelet proBDNF/BDNF ratio as a determinant of platelet biology. How the platelet proBDNF/BDNF ratio relates to neuronal levels, and whether platelets could be used as non-invasive biomarkers of neuronal health, remain open questions.

## Data Availability Statement

The raw data supporting the conclusions of this article will be made available by the authors, without undue reservation.

## Ethics Statement

The studies involving human participants were reviewed and approved by Montreal Heart Institute Scientific and Research Ethics Committee. The patients/participants provided their written informed consent to participate in this study.

## Author Contributions

JL has performed assays and collected data, analyzed and interpreted data, and wrote the manuscript. SF, IB, J-CB, and MW have performed assays and collected data, analyzed and interpreted data, and critically revised the manuscript. ML has overseen the research group, designed the research, obtained funding, analyzed and interpreted data, and critically revised the manuscript. All authors contributed to the article and approved the submitted version.

## Funding

This work was supported by the Canadian Institutes of Health Research (PJT-159569), the Canada Foundation for Innovation Leaders Opportunity Fund (32797), and the Montreal Heart Institute Foundation. JL was supported by summer internships from the Faculté de pharmacie of the Université de Montréal. SF was supported by scholarships from the Faculté de pharmacie, from the Faculté des études supérieures et postdoctorales of the Université de Montréal, from the Montreal Heart Institute Foundation and is a Canadian Vascular Network Scholar. IB was supported by scholarships from the Faculté de pharmacie, from the Faculté des études supérieures et postdoctorales of the Université de Montréal and from the Montreal Heart Institute Foundation. ML is a Fonds de recherche du Québec en Santé (FRQS) Junior 1 Research Scholar (33048). The funding bodies played no role in the design of the study, collection, analysis, and interpretation of data, or in writing the manuscript.

## Conflict of Interest

ML has received speaker fees from Bayer; has participated in industry-funded trials from Idorsia; has served on advisory boards for Servier; and has received in-kind and financial support for investigator-initiated grants from Leo Pharma, Roche Diagnostics, Aggredyne, and Fujimori Kogyo.

The remaining authors declare that the research was conducted in the absence of any commercial or financial relationships that could be construed as a potential conflict of interest.

## Abbreviations

AA, arachidonic acid; ACD, acid citrate dextrose; ADP, adenosine diphosphate; BDNF, brain-derived neurotrophic factor; BSA, bovine serum albumin; Ctrl, control; DABCO, 1,4-diazabicyclo(2,2,2)octane; EDTA, ethylenediaminetetraacetic acid; ELISA, enzyme-linked immunosorbent assay; IB, immunoblotting; IQR interquartile range; kDa, kilodaltons; PBMC, peripheral blood mononuclear cell; PBS, phosphate buffered saline; PFA, paraformaldehyde; PPP, platelet-poor plasma; proBDNF, pro-brain-derived neurotrophic factor; PRP, platelet-rich plasma; RIPA buffer, radioimmunoprecipitation assay buffer; rProBDNF, recombinant pro-brain-derived neurotrophic factor; RT, room temperature; SD, standard deviation; SDS, sodium dodecyl sulfate; SDS-PAGE, sodium dodecyl sulfate polyacrylamide gel electrophoresis; TBS-T, tris buffered saline-tween; TRAP, thrombin-related activating peptide; Tris, tris(hydroxymethyl)aminomethane.
