# Supplementary material for: Platelets Selectively Regulate the Release of BDNF, But Not That of Its Precursor Protein, proBDNF
Source: Front Immunol. 2020 Nov 25;11:575607. doi: 10.3389/fimmu.2020.575607 (PMC7723927; doi:10.3389/fimmu.2020.575607)
Supplement: Supplementary file 1 [file DataSheet_1.pdf]

Supplemental material to:

Platelets selectively regulate the release of BDNF, but not that of its precursor protein, proBDNF

Jessica Le Blanc[1,2], Samuel Fleury[1,2], Imane Boukhatem[1,2], Jean-Christophe Bélanger[1,2],

Mélanie Welman[2], Marie Lordkipanidzé[1,2,*]

[1] Faculty of Pharmacy, Université de Montréal, Montréal, Québec, Canada;
[2] Research Center, Montreal Heart Institute, Montréal, Québec, Canada;

*** Correspondence:**
Marie Lordkipanidzé
marie.lordkipanidze@umontreal.ca

proBDNF ELISA validation in plasma and lysis buffer

1. Supplementary Methods:

1.1 Sample preparation

To verify whether the proBDNF ELISA assay could safely be used in plasma samples and in lysis buffer, we replaced the manufacturer's recommended reagent diluent with either plasma diluted 1:15 in reagent diluent, or lysis buffer diluted 1:2 with reagent diluent. These dilutions were based on dilutions required to obtain workable concentrations with either plasma or platelet cell lysates for proBDNF in our cohort.

1.2 Standard curve

The standard curve was produced with the high-quality proBDNF recombinant protein from R&D systems, as supplied with the assay. The following concentrations were obtained through serial dilutions to generate the standard curve 5000, 2500, 1250, 625, 312.5, 156.25, 78.125, 0 (blank) pg/ml. Each condition was tested in duplicate. Colorimetric reading was performed with the Infinite F50 plate reader (Tecan, Männedorf, Switzerland) at 450 nm, with a reference at 620 nm.

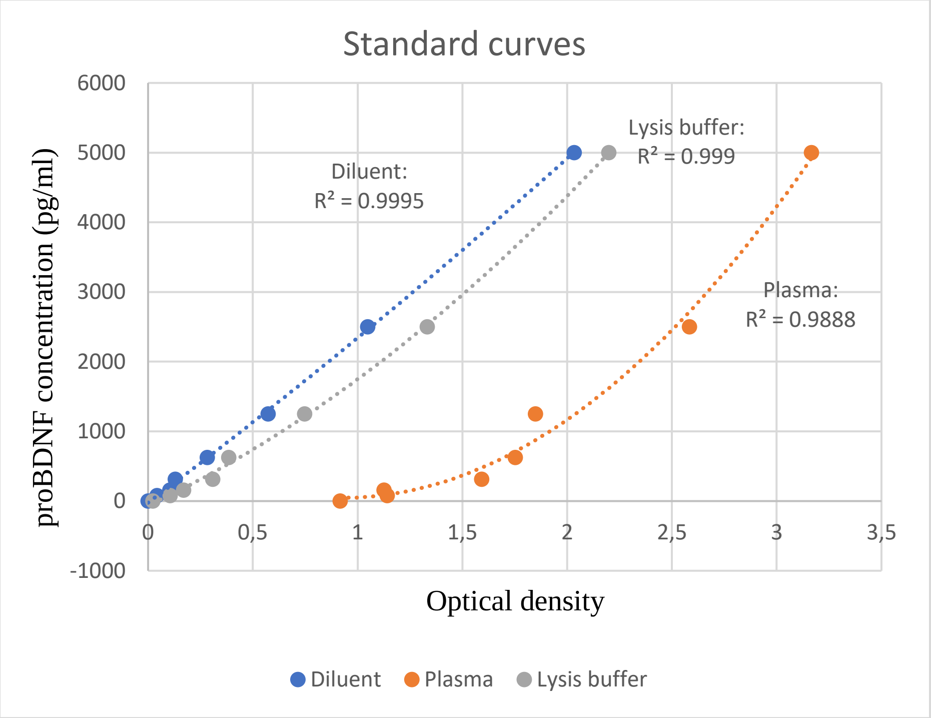

S-Figure 1: Standard curves in reagent diluent, lysis buffer and plasma. The diluent and lysis buffer gave similar results once fitted with a polynomial curve, with minimal differences in blank values and overall curve fitting. Plasma values were higher, due to the presence of endogenous proBDNF in samples. However, the standard curve followed expected increases in optical density with each proBDNF standard concentration increase.

1.3 Linearity in plasma concentrations

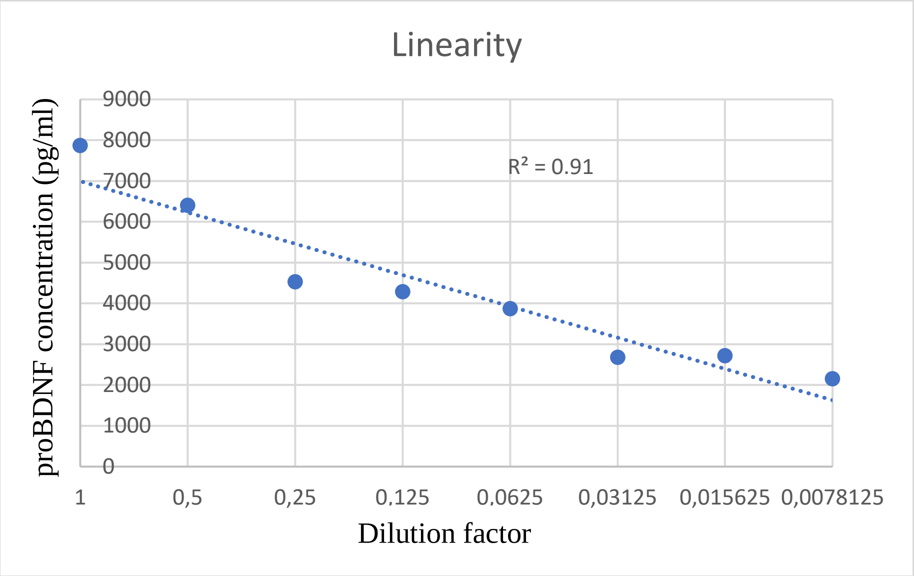

S-Figure 2: Linearity of serial dilutions in plasma.
To check for linearity of the association despite presence of endogenous proBDNF in the plasma samples, the observed proBDNF concentration were blotted against each serial two-fold dilution of the standard curve (R^2=0.91).

Specificity of R-176 and Mab31751 proBDNF antibodies

2. Supplementary Methods:

2.1 Cell culture and transfection

HEK293T cells were cultivated in 6 cm diameter Petri dishes in Dulbecco's Modified Eagle Medium (containing 10% fetal bovine serum, 1% penicillin-streptomycin) until confluence of 80% and were then transfected with C-terminal Myc-tagged proBDNF plasmid (proBDNF gene cloned in pCMV6-Entry Vector) (3 µg), using PolyJet reagent (9 µL). HEK293T cells treated with PolyJet DNA transfection reagent without DNA were used as negative controls. Culture medium was changed 24h after transfection. Cells were collected and lysed with RIPA buffer containing protease inhibitors, 48h post-transfection.

2.2 Immunoblotting

Immunoblotting was performed as described in section "*2.5 ProBDNF immunoblotting*". The following antibodies were used:

- proBDNF antibody, Biosensis, R-176 polyclonal rabbit antibody, 0.25 µg/ml
- proBDNF antibody, R&D systems, mab31751 monoclonal mouse antibody, clone 584412, 0.5 µg/ml
- Myc tag antibody, Novus, NB600-302, monoclonal mouse antibody, 2 µg/ml
- β-actin antibody, R&D systems, MAB8929, monoclonal mouse antibody, clone 937215, 10 ng/ml

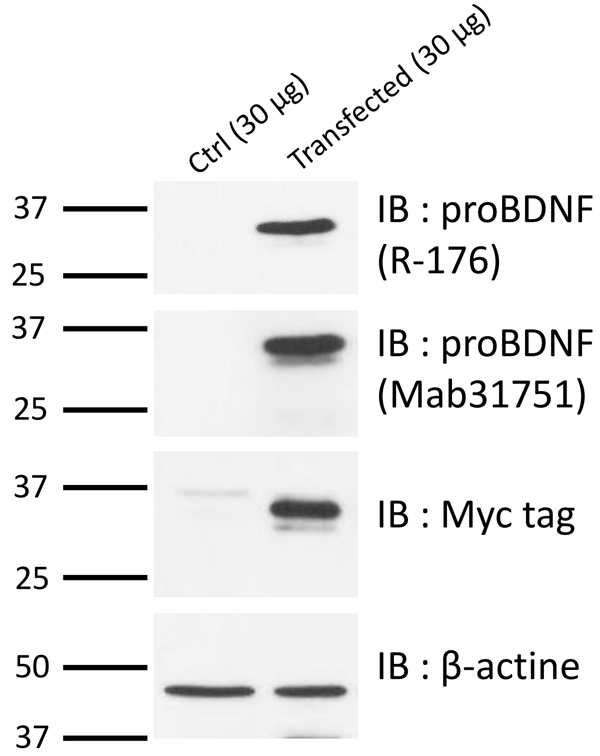

S-Figure 3. Specificity of R-176 and Mab31751 proBDNF antibodies. ProBDNF immunoblotting of HEK293T cells transfected with C-terminal Myc-tagged proBDNF plasmid to confirm the specificity of both proBDNF primary antibodies tested. HEK293T treated with PolyJet DNA transfection reagent without DNA were used as negative controls (Ctrl). ß - actin was used as loading control. Molecular weight is indicated on the left (kDa) and primary antibody on the right. n=2 independent transfections. IB: immunoblotting.

3. Supplementary Methods:

3.1 Acid wash experiments

Washed platelets were prepared as described in Method section “*2.2 Blood collection and platelet isolation*”. Acid washes were then performed using two different acid solution:

- Acid wash solution 1: 20 mM citrate, 0.15 M NaCl, 0.03 % w/v BSA, pH 4.5
- Acid wash solution 2: 0.2 M citric acid, 0.5 M NaCl, 0.2% w/v BSA, pH 2.67

Platelets were resuspended in one of the acid wash solutions containing prostaglandin E_1 (1 µM) for 5 minutes at RT with magnetic stirring. Then, platelets were centrifuged at 800 g for 8 minutes, the acid solution was removed and the pellet was resuspended in Tyrode’s buffer. Following a second centrifugation (800 g for 8 minutes), Tyrode’s buffer was removed and the platelet pellet was either lysed with RIPA buffer containing protease inhibitors for further immunoblotting analyses, or resuspended in Tyrode’s buffer for flow cytometry analyses.

3.2 Flow cytometry

Platelets were fixed with 1% paraformaldehyde (PFA) for 20 minutes at RT, and a fraction was also permeabilized using 0.1% Triton-X for 15 minutes at RT. Immunofluorescence labeling was performed (antibodies against proBDNF: R&D System, mab31751, monoclonal mouse antibody, clone 584412, and Biosensis, R-176 polyclonal rabbit antibody) as described in section “*2.6 Flow cytometry*”.

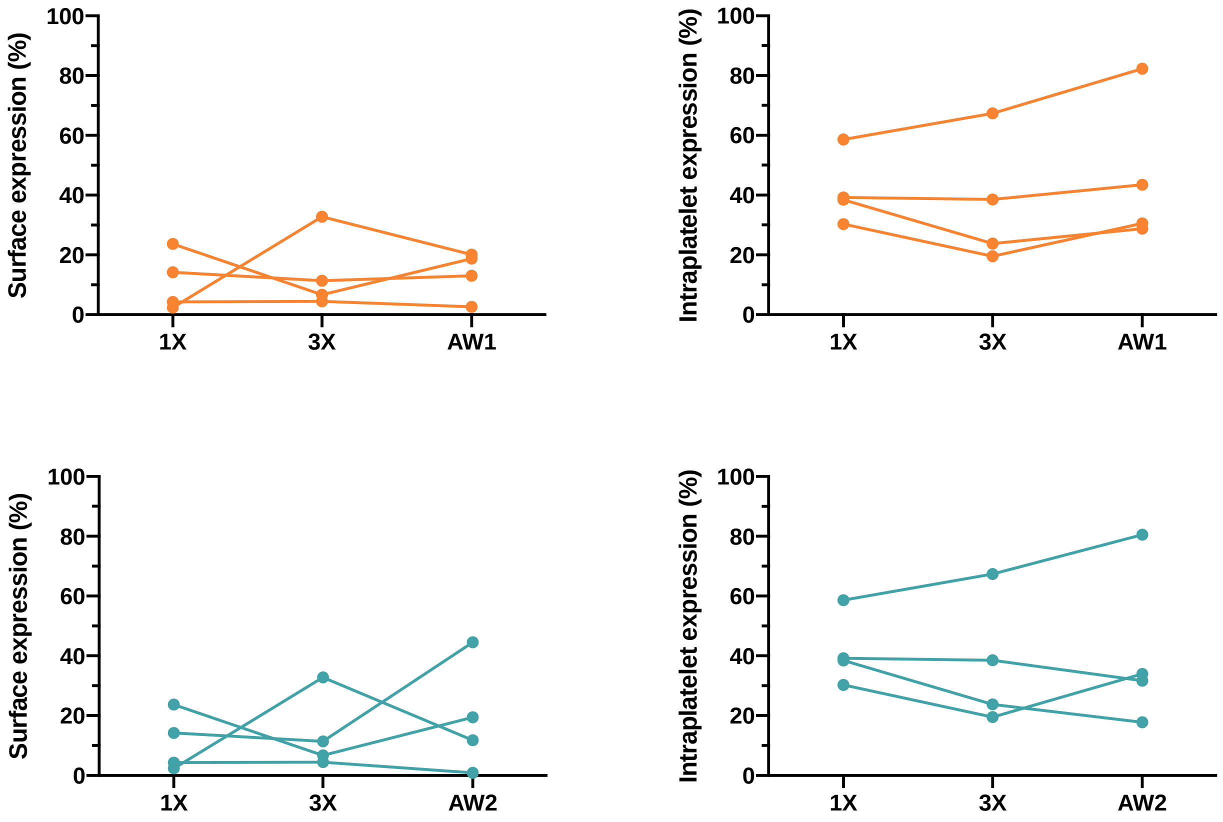

S-Figure 4. Washed platelet lysates are not contaminated by plasma proteins. Surface and intraplatelet expression of proBDNF obtained by flow cytometry after washing platelets with two different acid solutions to remove residual plasma proteins bound to membranes. Connected points represent one healthy volunteer (n=4). 1X: Platelets washed once with Tyrode's buffer as described in the platelet isolation section of the methods; 3X: Platelets washed 3 times with Tyrode's buffer; AW1: Platelets washed with acid wash solution 1; AW2: Platelets washed with acid wash solution 2.
